# A Case Report of a Giant Tubular Adenoma With a Concurrent Fibroadenoma of the Breast

**DOI:** 10.14740/wjon954e

**Published:** 2015-12-31

**Authors:** Sasank Kalipatnapu, Vimalin Samuel, Martha Johnson, Koshy Perookavil Daniel

**Affiliations:** a Department of General Surgery, Christian Medical College, Vellore, Tamilnadu, India; b Department of Pathology, Christian Fellowship Hospital, Oddanchatram, Dindigul District, Tamilnadu, India; c Department of Surgery, Christian Fellowship Hospital, Oddanchatram, Dindigul District, Tamilnadu, India

**Keywords:** Giant tubular adenoma, Fibroadenoma, Benign, Rare, Breast lump, Breast

## Abstract

Tubular adenomas are rare benign epithelial tumors of the breast. Only a handful of cases have been reported in literature. We describe a very rare case of a giant tubular adenoma with a concurrent fibroadenoma in a young woman.

## Introduction

Tubular adenomas are rare benign tumors of the breast seen predominantly in young women with only two cases of giant tubular adenoma being reported so far in literature [1-3]. Here, we report a case of a young woman with a giant tubular adenoma and a fibroadenoma in the same breast.

## Case Report

A 23-year-old woman presented with complaints of gradually enlarging painless lump in the left breast for 3 years. She had also noticed a new lump which had developed next to the previous lump for 6 months. She did not give history of any nipple discharge or cyclical mastalgia. Her menstrual cycles were regular and she did not have any other known comorbidities. She did not have any family history of similar illness or breast malignancies. On physical examination, there was an 8 × 10 cm firm lump with nodular surface, occupying the left breast between 2 and 6 o’clock position. A 2 × 3 cm firm mobile lump was also felt at 7 o’clock position on the same side. The skin overlying the swellings was normal. There was no palpable axillary or supraclavicular lymph nodes. A firm, mobile painless lump of 2 × 2 cm was palpable at 8 o’clock position in the contralateral breast. She was clinically suspected to have bilateral fibroadenomas. Due to the history of progressive enlargement of the left-sided lesion, she underwent an excision biopsy of both the lumps in her left breast. The patient opted not to have the surgery on the right side in the same setting. Postoperatively, her recovery was uneventful.

### Pathological features

Two encapsulated fibro-fatty masses were excised measuring 9.5 × 8.5 × 5 and 4 × 2 × 1 cm. Cut section of the larger mass showed grayish white and grayish yellow solid areas, while the smaller one had a grayish white surface with slit like areas. Under the microscope, the larger mass had closely packed tubular structures lined by single layer of secretory cells and few flattened myoepithelial cells, with scanty connective tissue stroma between the tubules. The smaller mass showed a biphasic tumor with proliferation of both glandular and stromal components. Based on their characteristic features, the larger swelling was identified to be a tubular adenoma while the smaller one was reported as a fibroadenoma.

This case is being reported in view of the rarity of occurrence of both tubular adenoma and fibroadenoma together. To the authors’ knowledge, there has been only one other case reported to date where both the histologies have been reported in close proximity [4].

## Discussion

Tubular adenoma, also called pure adenoma, is a rare benign epithelial tumor of the breast [1, 5]. They are a subclass of breast adenomas and are similar to pericanalicular fibroadenomas and they are usually diagnosed as a fibroadenoma due to their clinical and radiological similarities [6]. They are seen predominantly in young women or those in reproductive age group and are very rare in the elderly age group with only two cases of postmenopausal tubular adenoma having been reported in literature [1, 7]. The risk of malignant transformation is almost non-existent, with only one case of probable transformation having been reported in literature [8]. They usually present as painless freely mobile well-circumscribed masses ranging in size from 1 cm to over 7.5 cm [1]. On histological examination, they show the presence of tightly packed epithelial elements (predominantly acini and tubules) with only minimal intervening stroma [1, 6]. Based on these features, it has been suggested that they may be related histogenetically to fibroadenomas [1]. On radiological examination, they appear remarkably similar to non-calcified fibroadenomas in young women. However, in older women, they have been shown to exhibit microcalcifications which could be misinterpreted as malignancy. Dense or irregular microcalcifications seen within the mass in a tightly grouped manner could point towards the diagnosis of a tubular adenoma [6]. The differentiation can be definitively made on histopathology and complete excision of the lump is curative [9].

### Conclusion

Tubular adenomas are rare benign lesions which could potentially be confused with fibroadenomas due to their similarity on preoperative evaluation. The development of a tubular adenoma along with a fibroadenoma in the same breast concurrently, as seen in our case, is a very rare occurrence. Surgical excision and histopathological examination is required for accurate diagnosis. Although tubular adenoma is rare, it should always be considered in the differential diagnosis for a benign lump in the breast.
